# Continuous Transmission Frequency Modulation Detection under Variable Sonar-Target Speed Conditions

**DOI:** 10.3390/s130303549

**Published:** 2013-03-13

**Authors:** Yang Wang, Jun Yang

**Affiliations:** 1 Key Laboratory of Noise and Vibration Research, Institute of Acoustics, Chinese Academy of Sciences, Beijing 100190, China; E-Mail: wangyang1@mail.ioa.ac.cn; 2 The State Key Laboratory of Acoustics, Institute of Acoustics, Chinese Academy of Sciences, Beijing 100190, China

**Keywords:** CTFM, ultrasonic ranging sensor, Doppler shift, deviation, correction, range profile

## Abstract

As a ranging sensor, a continuous transmission frequency modulation (CTFM) sonar with its ability for range finding and range profile formation works effectively under stationary conditions. When a relative velocity exists between the target and the sonar, the echo signal is Doppler-shifted. This situation causes the output of the sensor to deviate from the actual target range, thus limiting its applications to stationary conditions only. This work presents an approach for correcting such a deviation. By analyzing the Doppler effect during the propagation process, the sensor output can be corrected by a Doppler factor. To obtain this factor, a conventional CTFM system is slightly modified by adding a single tone signal with a frequency that locates out-of-sweep range of the transmitted signal. The Doppler factor can be extracted from the echo. Both verification experiments and performance tests are carried out. Results indicate the validity of the proposed approach. Moreover, ranging precision under different processing setups is discussed. For adjacent multiple targets, the discrimination ability is influenced by displacement and velocity. A discrimination boundary is provided through an analysis.

## 1. Introduction

Ultrasonic ranging sensors have been widely used in collision avoidance [1], navigation [2,3], mapping [4,5], target classification [6,11], and in travel aid devices for visually impaired individuals [7,8]. The ultrasonic ranging sensor, which is low cost, accurate in positioning, and easy to operate, is considered as a competitive choice for implementing target detection and recognition. Sensors that work in pulse-echo mode, which is the most common mode, have been applied in various forms [9,10]. However, most pulse-echo sensors only retain the return of the nearest target and neglect the rest of the echo signals.

A more sophisticated ultrasonic sensor, a continuous transmission frequency modulation (CTFM) sonar, has been extensively developed in the past several decades. With its advantages of high precision, broadband, high signal-to-noise ratio, and high quantity of information, the CTFM sonar is capable of detecting multiple targets, classifying primitive indoor targets [11], and even recognizing complex targets such as rough surfaces [12], leafy plants [13], and human faces [14]. However, most of the applications of a CTFM sonar are carried out under stationary conditions. When one or both of the sonar and the target are moving, the echo signal is Doppler-shifted. After demodulation, the output of the system is deviated from the actual target range. This situation limits the application of a CTFM sonar to stationary platforms and motionless-target sensing.

An early study of a CTFM sonar provided an analysis of how the system output changes with a moving target [15]. In a more recent work, a CTFM sonar was mounted on a mobile agent to classify different rough surfaces [16]. The spectrum of the demodulation output signal was averaged to reduce the influence of Doppler shift to the range profile. However, the output range value deviation was not considered. Until now, no effective solution has been provided to eliminate the aforementioned deviation. This work presents an approach for correcting CTFM output under non-stationary conditions with a slight modification to the system. With this approach, the system still works with high precision, and is now capable of generating the range profile of complex targets. The modified system can be further applied to non-stationary scenarios such as robot navigation and moving-target detection.

In the following section, a description is provided on how a conventional CTFM sonar system works. A dual-sweep demodulation approach is introduced to eliminate the “blind time” in the output signal of a conventional CTFM sonar system. Noise and interference that may affect the system are discussed. In Section 3, a general condition of the propagation procedure of the ultrasonic signal between a moving sonar and a moving target is analyzed. A Doppler factor is introduced to correct the system output. Section 4 presents experimental studies for verification and performance tests for the proposed approach. A detailed discussion of the experiment results and the system performance is given in Section 5. Finally, conclusions and future research considerations are summarized in Section 6.

## 2. CTFM System Description

### 2.1. Basic CTFM System

A basic CTFM system transmits a cyclic linear sweep signal *S_T_*(*t*). In one sweep period, *S_T_*(*t*) can be described as:

(1) $$\begin{array}{ll} S_{T}(t)=A\sin\left[2\pi \left(f_{H}-\frac{m}{2}t\right)t\right] & \left(0<t<T_{sw}\right) \end{array}$$

*S_T_*(*t*) sweeps from *f_H_* down to *f_L_* during a period of *T_sw_*. The sweep bandwidth is *B* = *f_H_* − *f_L_*, and the sweep rate is *m* = *B*/*T_sw_*. *A* is the signal amplitude. The echo signal is a replica of *S_T_* with a time delay of:

(2) τ=2R/c

where *R* is the range to the target, and *c* is the sound speed. The echo signal can be described as:

(3) $$S_{R}(t)=A\beta (\lambda ,R,\theta )S_{T}(t-\tau )$$

where *β*(*λ*,*R*,*θ*) is an attenuation factor related to the wavelength *λ*, the range *R*, and the incident angle *θ*. The amplitude of the echo also depends on the surface characteristic of the reflector [6,12]. To focus on the range finding function of the system, it is assumed that the targets are strong reflectors and are within the observable area.

The frequency of *S_T_*(*t*) is:

(4) $$\begin{array}{ll} f_{T}(t)=\frac{1}{2\pi }\frac{d\Phi_{T}(t)}{dt}=f_{H}-mt & \left(0<t<T_{sw}\right) \end{array}$$

where *Φ_T_*(*t*) = *2π*(*f_H_* − *mt*/2)*t* is the instantaneous phase of *S_T_*(*t*). Similarly, the frequency of *S_R_*(*t*) is:

(5) $$f_{R}(t)=f_{T}(t-\tau )=f_{H}-m(t-\tau )$$

The demodulation procedure includes mixing and filtering operations. When *S_T_*(*t*) is mixed with *S_R_*(*t*), a sum frequency and a difference frequency component are included in the product signal. The sum is filtered out by a low-pass filter (LPF), the difference frequency component *f_α_* is left as the demodulation output signal:

(6) $$f_{\alpha }=m\tau =\frac{2Rm}{c}$$

Therefore, the measured range value can be written as:

(7) $$R=\frac{cf_{\alpha }}{2m}$$

**Figure 1.**
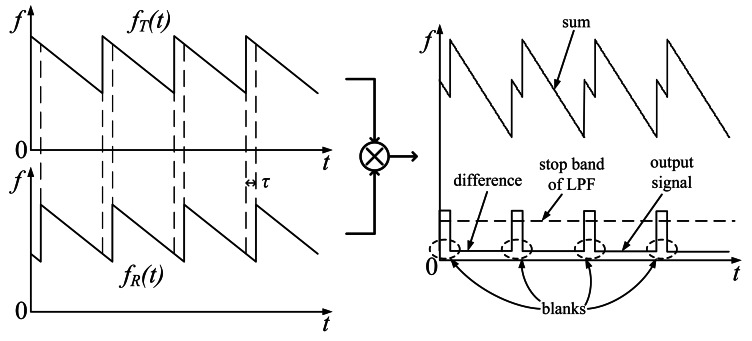
The signal spectrogram of the demodulation procedure.

A spectrogram explanation is shown in Figure 1. The component *f_α_* is obviously proportional to range *R*. A reflector can be seen as a peak in the spectrum of the demodulation output signal. If the echo comes from multiple reflectors, corresponding peaks can be found in the spectrum. In a CTFM sonar system, signal frequency is concerned with target range. Therefore, all the analyses in this paper focus only on the frequency domain.

### 2.2. Dual Sweep Demodulation

The output of a basic CTFM system includes a blank in every sweep period. The blank appears at the start of a sweep period with a length of *τ*. Such blanks are called “blind time,” they make the output discontinuous and decrease output signal energy. A dual demodulation method has been brought out to eliminate blind time [17]. An additional sweep signal, with a sweep rate equal to *m* and a band next to the band of *S_T_*(*t*), is introduced in the demodulation procedure. The additional sweep signal is described as:

(8) $$\begin{array}{ll} S_{A}(t)=A\sin\left[2\pi \left(f_{L}-\frac{m}{2}t\right)t\right] & \left(0<t<T_{sw}^{\prime }\right) \end{array}$$

Similar to Equation (4), the frequency of *S_A_*(*t*) is:

(9) $$\begin{array}{ll} f_{A}(t)=\frac{1}{2\pi }\frac{d\Phi_{A}(t)}{dt}=f_{L}-mt & \left(0<t<T_{sw}^{\prime }\right) \end{array}$$

where *Φ_A_*(*t*) = *2π*(*f_L_* − *mt*/2)*t* is the instantaneous phase of *S_A_*(*t*).

The echo signal mixes with the sum signals of *S_T_*(*t*) and *S_A_*(*t*) instead of *S_T_*(*t*) only. Output signal frequency can be written as:

(10) $$f_{\alpha }(t)=\{\begin{array}{cc} f_{R}(t)-f_{A}(t) & 0<t<\tau \\ f_{R}(t)-f_{T}(t) & \tau <t<T_{sw} \end{array}$$

**Figure 2.**
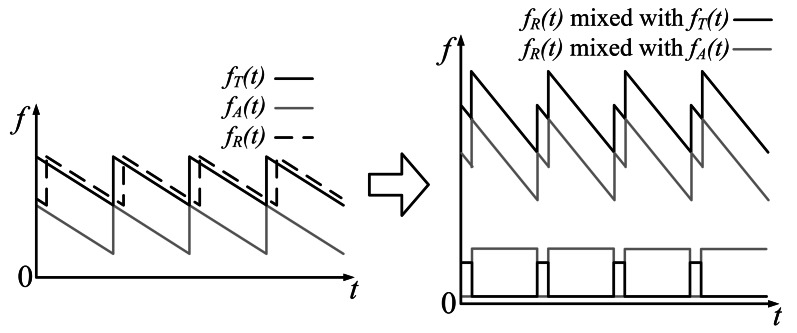
Signal spectrogram of a dual demodulation system.

*S_A_*(*t*) can be considered as an extension of *S_T_*(*t*) in the spectrogram. In such a manner, *S_A_*(*t*) forms a sweep signal with a period long enough to cover the echo signal; therefore, blind time gaps are filled. In practice, *T*′*_sw_* is set to be equal to *T_sw_*, thus *S_A_*(*t*) shares the same trigger signal with *S_T_*(*t*) to reduce the complexity of signal generation (Figure 2).

Note that the maximum value of the difference frequency between *f_R_*(*t*) and *f_T_*(*t*) is *B*/2, which corresponds to a range value of *cT_sw_*/4. A reflector farther than this value will make *f_R_*(*t*) demodulated with *f_T_*(*t*) in the next sweep period. Targets around *cT_sw_*/4 cause range ambiguity or phantom targets. To avoid this situation, firstly the stop band of the LPF is usually set lower than *B*/2 to eliminate range ambiguity. Furthermore, the intensity of the transmitted signal can be adjusted to an appropriate value, so that the echo reflected from farther than *cT_sw_*/4 dies out before it reaches back to the sonar. Actually, in most airborne ultrasonic uses, phantom target seldom appears due to the rapid attenuation of the ultrasonic signal. Therefore only the first step is needed to be done in the system design, and only targets found within the region of 0 ∼ *cT_sw_*/4 are meaningful.

### 2.3. Noise and Interference

Different from the pulse-echo sonar, the CTFM sonar operates in a continuous manner, which provides a much higher power output. Meanwhile, the dual sweep demodulation operation fills the blind time of the output signal, which further enhances the energy of the output. The demodulation procedure transfers the echo signal in time domain to frequency domain for indication. The target can be seen in the spectrum of the output signal generated using Fourier Transform. The Fourier Transform also provides an averaging process that reduces the noise. A band-pass filter (BPF) applied to the echo signal can eliminate the noise out of the sweep band. The pass band of the BPF should be designed wider than the sweep band of the transmitted signal. The margins on both sides of the pass band are left for the Doppler shifted echo signal.

Although CTFM sonar has the ability of noise suppression, crosstalk between the transmission and the reception channel still exists. The transmitted signal may mix into the received echo signal, which appears as a large reflector very close to the sonar. The impact of this interference can also be eliminated by filtering. Instead of a LPF, another BPF can be applied following the mixing operation in the demodulation procedure to filter out the interference and the sum frequency.

**Figure 3.**
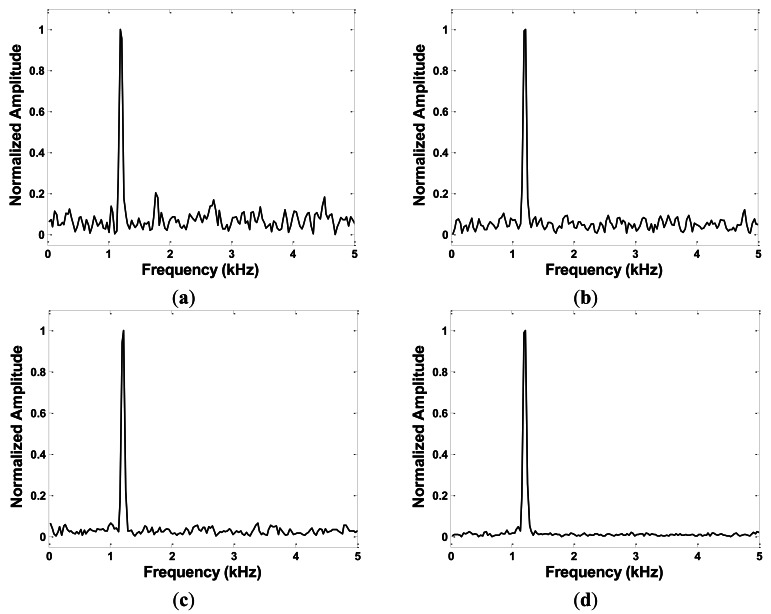
Output signal spectrum under echo SNR of (**a**) 0 dB; (**b**) 3 dB; (**c**) 10 dB; and (**d**) 20 dB.

The filters guarantee a high quality of the output signal in most practical uses. However, under harsh signal conditions, the system might be disturbed by unknown noise that cannot be ignored. Figure 3 shows the spectrum of the output signal when the echo signal is affected by additive white Gaussian noise. These results are obtained under assumptions that the reflector is 1 m away from the sonar, and the echo signal is amplified to the same magnitude of the sum signals of *S_T_*(*t*) and *S_A_*(*t*). The peak corresponding to the target still can be seen clearly, even when the echo signal has a signal-to-noise-ratio (SNR) of 0 dB. In the system described below, the BPFs are also applied, and the signal condition is considered to be good enough to generate a clear indication of the target.

## 3. Detection under Non-Stationary Conditions

Range detection can be quite accurate when the sonar and the target are both stationary. However, when a relative movement occurs between the target and the sonar, the echo is Doppler-shifted. As a result, the frequency of the demodulation output signal *f_α_* deviates from the actual target range value. The deviation increases along with relative velocity. In this case, detection result may easily become unacceptable.

### 3.1. Analysis of Ultrasonic Propagation

**Figure 4.**
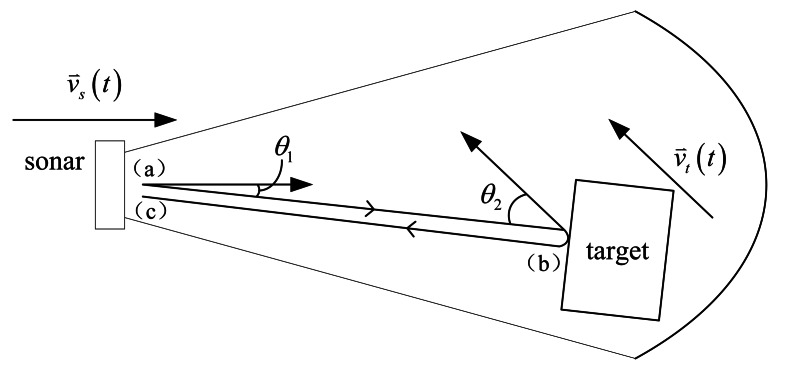
The propagation of the ultrasonic signal.

In general, the sonar and the target are both moving at different velocities and toward different directions. When the target is within the beam of the sonar, the Doppler effect occurs at three moments in the entire procedure of ultrasonic signal propagation, namely: (a) at transmission, (b) at reflection, and (c) at reception, as shown in Figure 4.

Supposing that an arbitrary signal with an instantaneous frequency of *f*(*t*) is transmitted by the sonar, then, only the ultrasonic beam that hits the target and is reflected back to the sonar is considered. The sonar is moving at a velocity vector of $\vec{v_{s}}(t)$ at the moment of transmission. The angle between $\vec{v_{s}}(t)$ and the beam is *θ_1_*. After transmission, *f*(*t*) is shifted to*f̂*_1_(*t*) because of the Doppler effect (a) [18]:

(11) $${\hat{f}}_{1}(t)=f(t)\frac{c}{c-v_{s}(t)\cos\theta_{1}}$$

After traveling for a time of flight (TOF) of *τ_1_*(*t*), the signal hits the target. The target is moving at a velocity vector of $\vec{v_{t}}(t)$ with an angle of *θ_2_* from the beam, at the moment of reflection. Because of the Doppler effect (b), the frequency of the echo becomes:

(12) $${\hat{f}}_{2}(t)={\hat{f}}_{1}(t-\tau_{1}(t))\frac{c+v_{t}(t)\cos\theta_{2}}{c-v_{s}(t)\cos\theta_{2}}$$

After traveling once more for *τ_2_*(*t*), the signal returns to the sonar. At the moment of reception, the total TOF of the signal is the sum of *τ_1_*(*t*)and *τ_2_*(*t*): *τ*(*t*) = *τ_1_*(*t*) + *τ_2_*(*t*). Assuming that *τ*(*t*) is short enough to neglect the displacement and the velocity change of the sonar, then the angle between $\vec{v_{s}}(t)$ and the echo beam is still *θ_1_*. As a result of the Doppler effect (c), the received signal frequency can be described as:

(13) $$\hat{f}(t)={\hat{f}}_{2}(t-\tau_{2}(t))\frac{c+v_{s}(t)\cos\theta_{1}}{c}$$

By substituting Equation (11) into Equation (12), and then into Equation (13), *f̂*(*t*) can be described by *f*(*t*), that is:

(14) $$\hat{f}(t)=f(t-\tau (t))\frac{c+v_{s}(t)\cos\theta_{1}}{c-v_{s}(t-\tau (t))\cos\theta_{1}}\frac{c+v_{t}(t-\tau_{2}(t))\cos\theta_{2}}{c-v_{t}(t-\tau_{2}(t))\cos\theta_{2}}$$

Examining Equation (14) shows that after the entire propagation in a period of *τ*(*t*), the signal is shifted from the transmitted signal *f*(*t-τ*(*t*)) to the received signal *f̂*(*t*) by a Doppler factor *D*(*t*), which is the total shift ratio, that is:

(15) $$\hat{f}(t)=f(t-\tau (t))D(t)$$

According to Equations (14) and (15), *D*(*t*) is:

(16) $$D(t)=\frac{c+v_{s}(t)\cos\theta_{1}}{c-v_{s}(t)\cos\theta_{1}}\frac{c+v_{t}(t)\cos\theta_{2}}{c-v_{t}(t)\cos\theta_{2}}$$

If a single tone signal with a known frequency *f_ST_* is transmitted, *D*(*t*) can be easily acquired by:

(17) $$D(t)=\frac{{\hat{f}}_{ST}(t)}{f_{ST}}$$

where *f̂_ST_*(*t*) is the received signal frequency of the single tone signal.

### 3.2. Correction of the CTFM Output

The received signal frequency *f_T_* is a replica of the transmitted signal frequency shifted by *D*(*t*), that is:

(18) $${\hat{f}}_{T}(t)=f_{T}(t-\tau (t))D(t)$$

According to Equation (10), the frequency of the demodulation output signal is:

(19) $${\hat{f}}_{\alpha }(t)=\{\begin{array}{cc} {\hat{f}}_{T}(t)-f_{A}(t) & 0<t<\tau (t)\\ {\hat{f}}_{T}(t)-f_{T}(t) & \tau (t)<t<T_{sw} \end{array}$$

**Figure 5.**
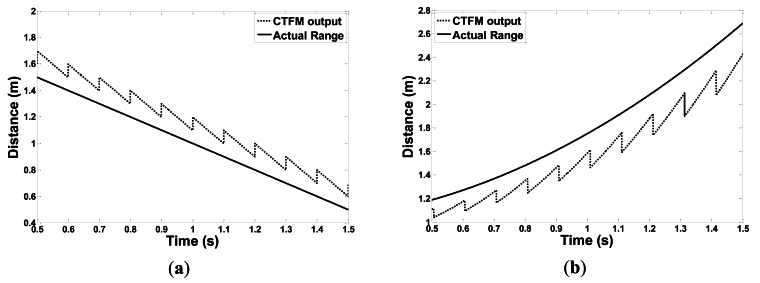
The CTFM output *vs*. the actual target distance. (**a**) A target approaching the sonar at 1 m·s^−1^; (**b**) A target accelerating away from the sonar at 1.5 m·s^−2^.

Obviously, *f̂*_α_ no longer follows Equation (6). Figure 5 demonstrates two moving conditions, deviations occur between the CTFM output and the actual target range. The CTFM output exhibits an oblique, sawtooth shape because higher velocity makes the Doppler factor *D*(*t*) larger, thus its contribution to the deviation is also greater, according to Equations (18) and (19). At the same time, a higher transmitted frequency also contributes more to the deviation.

To obtain a correct output, a single tone component *S_ST_*(*t*) with a frequency of *f_ST_* is added to the transmitted sweep signal *S_T_*(*t*). A blank frequency gap should be left between *f_ST_* and the sweep range of *S_T_*. This gap should be wide enough so that *f_ST_* and *f_T_*(*t*) will not interfere with each other after Doppler-shifting. The actual transmitted signal becomes:

(20) $$S_{T}^{\prime }(t)=S_{ST}(t)+S_{T}(t)$$

Because *f̂_ST_*, which is the frequency of Doppler-shifted *S_ST_* in the received signal, can be easily obtained, the Doppler factor *D*(*t*) is also obtained by using Equation (17). To acquire the actual range of the target, the aim is to determine the value of *f*_α_(*t*) that follows Equation (6). Thus, Equations (5), (18), and (19) are combined:

(21) $${\hat{f}}_{\alpha }(t)=\{\begin{array}{cc} \left[f_{\alpha }(t)+f_{A}(t)]D(t)-f_{A}(t\right) & 0<t<\tau (t)\\ \left[f_{\alpha }(t)+f_{T}(t)]D(t)-f_{T}(t\right) & \tau (t)<t<T_{sw} \end{array}$$

When Equation (21) is reorganized, *f*_α_(*t*) can be written as:

(22) $$f_{\alpha }(t)=\{\begin{array}{cc} \frac{{\hat{f}}_{\alpha }(t)+(1-D(t))f_{A}(t)}{D(t)} & 0<t<\tau (t)\\ \frac{{\hat{f}}_{\alpha }(t)+(1-D(t))f_{T}(t)}{D(t)} & \tau (t)<t<T_{sw} \end{array}$$

The target range can be solved using an equation similar to Equation (7):

(23) $$r(t-\tau (t))=\frac{cf_{\alpha }(t)}{2m}$$

Note that in Equation (22), all the components are available except for the total TOF *τ*(*t*), which acts as a dividing point of the equation. *τ*(*t*) can be calculated using *τ* = 2*r*/*c*. The range value at the end of a sweep period can be calculated using the second stage of Equations (22) and (23). In low-velocity conditions, *τ*(*t*) can be approximately calculated by *τ ≈* 2*r*(0)/*c*. In higher-velocity conditions, velocity or even acceleration can be introduced in estimating *τ*(*t*). Velocity and acceleration can be estimated using the previously acquired nearby range values. In this manner, all components that describe *f*_α_(*t*) are available. By integrating Equation (22) with Equation (23), the range value *r*(*t* − *τ*(*t*)) can be acquired.

## 4. Experiment1

The approach described in the previous section is verified and tested through experimental analysis. First, the equipment and the setup of the experiments are introduced below.

### 4.1. Experiment Setup

The pieces of equipment used in the experiment consisted of:

1. The sonar head is composed of a SensComp 600 instrumental transducer as the transmitter and a G.R.A.S. 46BE microphone as the receiver. The SensComp 600 is an electrostatic transducer with a beam angle of 15° at −6 dB.
2. The target is made up of boxes. One box only for a simple target, or more than one box put together to create a complex target with multiple reflectors.
3. Two identical slide rails are marked as A and B. The sonar head and the target are respectively fixed on the slider of slide rails A and B. The slider is driven by a stepper motor mounted on the end of the rail through a conveyor belt. The slider can reach a maximum speed of 1.6 m·s^−1^. The maximum travel length of the slide rails is 1 m.
4. Two amplifiers are used. One is for amplifying the echo signal received by the microphone; the other is for amplifying the transmitted signal and for driving the transducer.
5. An ADLINK MXC-6000 expandable computer is used. The computer is embedded with an ADLINK DAQ-2010 data acquisition card.

**Figure 6.**
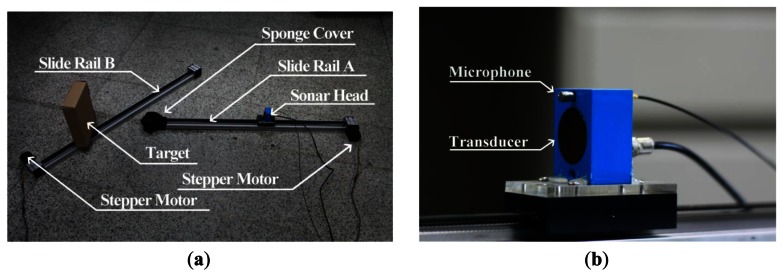
Pictures of the experimental apparatus. (**a**) The entire motion system; (**b**) The sonar head.

Pictures of the movement mechanism and the sonar head used in the experiments are shown in Figure 6.

**Figure 7.**
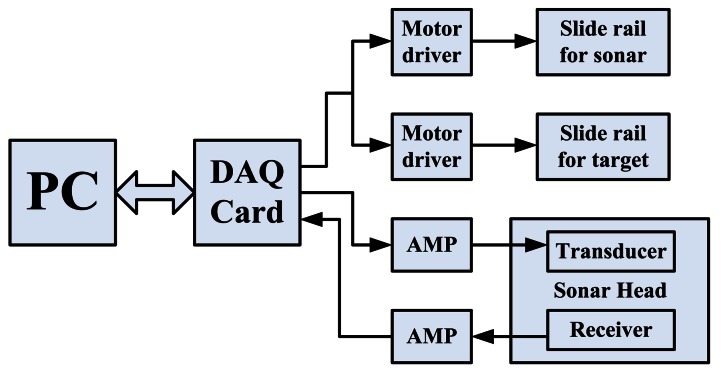
The control diagram of the experimental apparatus.

The connections among the apparatuses are shown in Figure 7. The data acquisition card is controlled by the computer through MATLAB software. Two of the analog output (AO) channels are used to export pulse trains to the stepper motors through two motor drivers. Another AO channel is used to export the transmitted signal to the sonar head. An analog input channel collects the echo signal from the sonar head. All the aforementioned signal operations are performed under a sample rate of 1 MS·s^−1^. Transmitted signal parameters are listed in Table 1.

**Figure 8.**
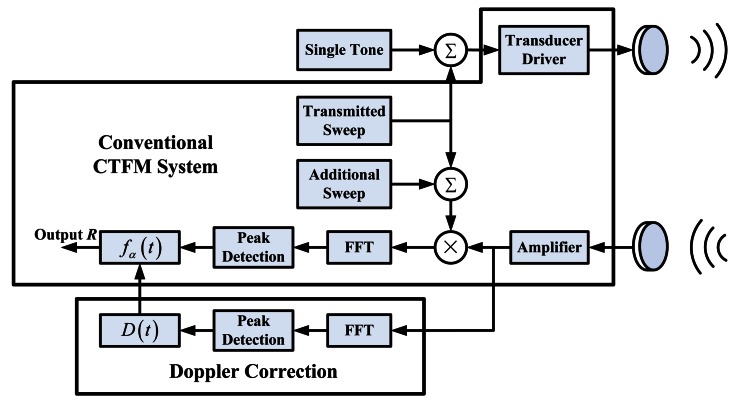
A block diagram of the system.

Transmission and reception of the CTFM signal and the motor control signal are performed at the same time. All input and output signals are stored in the computer. The demodulation operation is performed offline afterwards. First, signals are filtered to eliminate noise from the useful band. Signals are processed in frames. The spectrum of each frame is obtained by fast Fourier transform (FFT) analysis. The parameters *f̂_ST_*(*t*) and *f̂*_α_(*t*) can be determined by searching the peak in the spectrum of the echo signal and the demodulated signal. Thus, the Doppler factor *D*(*t*) and *f*_α_(*t*) can be determined using Equations (17) and (22), respectively. The target range can be obtained by substituting *f*_α_(*t*) into Equations (23). The processing procedure can be described as a block diagram, as shown in Figure 8.

The instantaneous positions of the sliders can be determined by the pulse number of the motor control signal. The initial geometric relationship between the two slide rails are measured manually. Actual range to the target at any moment can be acquired by trigonometric calculation.

### 4.2. Verification Experiments

**Figure 9.**
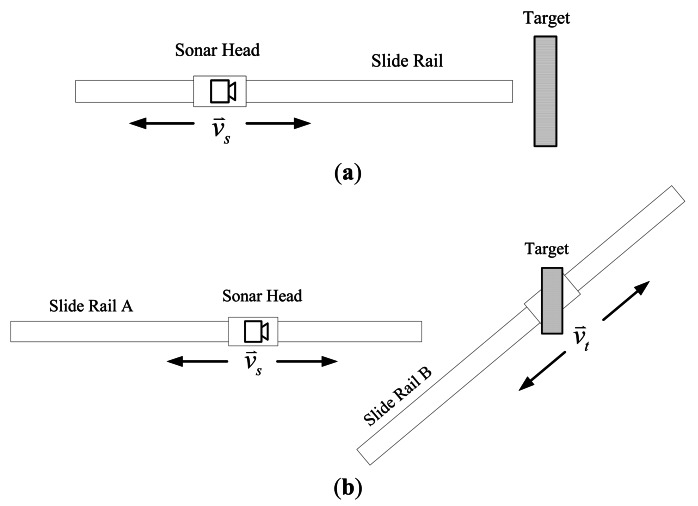
The setup for the two tested conditions. (**a**) The sonar head is moving while the target remains still; (**b**) Both the sonar head and the target are moving.

In the following experiments, a large frame with 16,384 samples and a short frame shift with 5,000 samples are set up. This setup produces a high frame rate to allow verification of the approach with dense data. Verification experiments are performed in two conditions: (1) the sonar is moving while the target remains still; the slide rail and the target are arranged as shown in Figure 9(a); (2) both the sonar and the target are moving, the two slide rails are arranged as shown in Figure 9(b).

**Figure 10.**
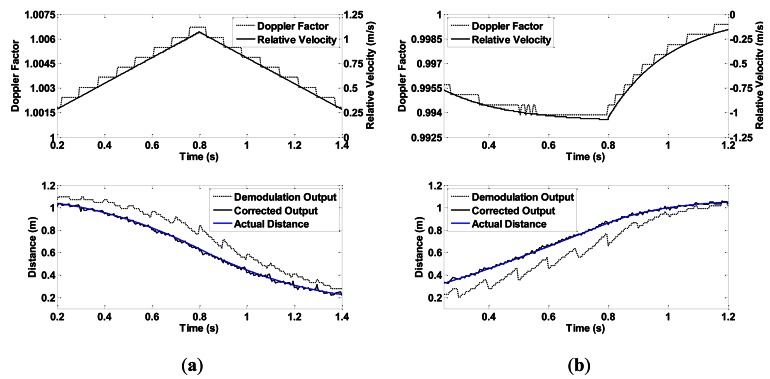
Experiment results of condition 1, the speed of the sonar head changes (**a**) linearly; and (**b**) exponentially.

In both conditions, the slider is controlled to speed up first, and then to slow down. The speeds of the sonar head and the target follow different acceleration curves to create various motions. In condition 1, the target is fixed close to the end of slide rail A. The sonar moves toward or away from the target. Figure 10 show experiment results when the speed of the sonar head follows a linear and an exponential curve, respectively.

**Figure 11.**
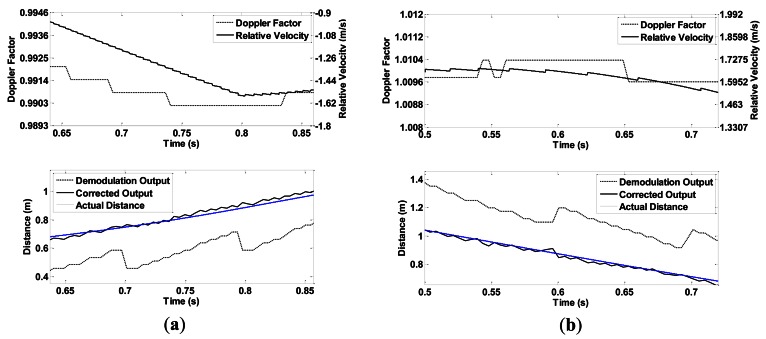
Experiment results of condition 2, the speeds of both the sonar head and the target change (**a**) linearly; and (**b**) exponentially.

In condition 2, the two slide rails make the same movement at the same time. As can be seen in Figure 9(b), an angle is formed between rail slides A and B. Therefore, the target is within the sonar beam only for a short time. Figure 11 shows two experimental results in which the sliders move at a linear velocity curve and an exponential curve.

### 4.3. Performance Tests

A series of constant speed experiments is performed to test the performance of the approach. The experimental setup is the same as that in condition 1, which is described in the previous subsection. The effect of correction is tested at different velocities and frame sizes.

**Figure 12.**
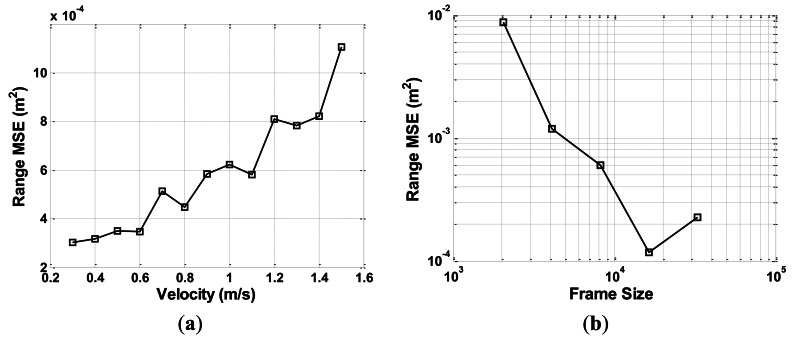
Results of performance tests (**a**) variance of corrected range *vs*. velocity and (**b**) variance of corrected range *vs.* frame size.

CTFM output range data take the shape of a step because of the discrete output of FFT analysis. After correction, data take a minor sawtooth shape. Higher velocity causes larger step difference, which leads to higher oscillation of the corrected range value. By contrast, a larger frame size produces smaller intervals between FFT outputs, thus leading to a higher range resolution. These inferences are supported by the experimental results shown in Figure 12.

Figure 12(a) indicates how the mean square error (MSE) of corrected range value changes with the velocity of the sonar movement. Thirteen velocity values from 0.3 m/s to 1.5 m/s are tested, with an interval of 0.5 m/s. Figure 12(b) indicates the relationship between the MSE of the corrected range and data frame size, under the velocity of 1 m/s. Five frame sizes, 2,048, 4,096, 8,192, 16,384, and 32,768, are tested. Frame shift is equal to half the frame size. In the aforementioned test results, each value point is an average of six tests at a corresponding velocity, with three approaching movements and three backing-away movements.

### 4.4. Complex Target Tests

Multiple reflectors distributed in different ranges produce a more complex spectrum. Such spectrum can be seen as a range profile that represents the geometric information of the target [13].

**Figure 13.**
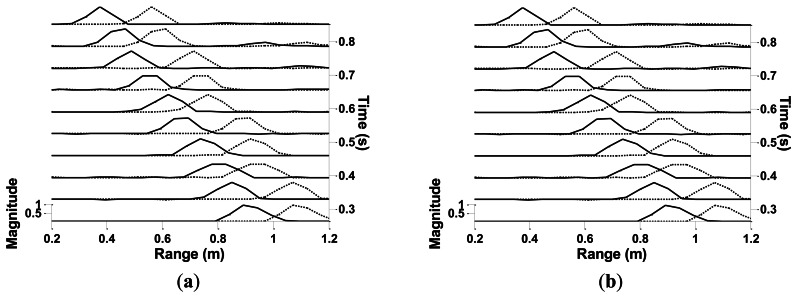

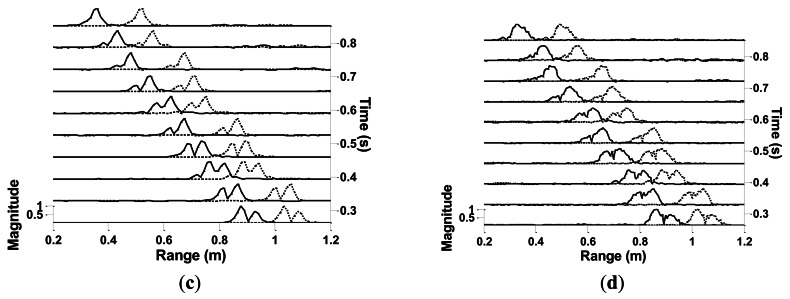
Results of range profile correction using different frame sizes: (**a**) 8,192; (**b**) 16,384; (**c**) 32,768; and (**d**) 65,536.

In this subsection, several initial complex target experiments are performed to support the discussion in Subsection 5.2. Two boxes are placed side by side in front of the sonar, which forms a two-reflector target. The sides facing the sonar are misaligned by 5 cm, thus creating two reflectors. The setup of the test is the same as that in condition 1 in Subsection 4.2. The velocity of the sonar is 1 m·s^−1^. Four frame sizes are chosen to be tested. The correction approach is applied to the whole range profile. Correction results are shown in Figure 13. The magnitude of the range profiles is normalized. The shifted and corrected range profiles are plotted in dashed and solid lines, respectively. In a stationary condition, the two reflectors form two corresponding peaks with a dip between them. In moving conditions, however, the two peaks may merge with each other or may become vague.

## 5. Discussion

### 5.1. Result Analysis

As discussed in Section 3, the deviation of the CTFM output is caused by Doppler shift when relative movement occurs between the sonar and the target. Supposing that the simultaneous motion information of the sonar and the target is available, then the frequency shift of the echo signal can be estimated through geometric calculation. Unfortunately, this information is unavailable in practice. Even if the motion of the mobile agent that carries the sonar can be obtained using velocity measurement devices such as encoders, angle values of the ultrasonic beam during the propagation will remain unknown. In this case, Doppler shift information obtained from the echo signal itself is the best match for the propagation procedure. However, in a CTFM system, signal frequency is changing all the time, thus Doppler shift is also hard to obtain. An idea that inspired the proposed approach is to combine the concepts of a CTFM sonar and a Doppler sonar. The sweep signal of a CTFM sonar and the single tone signal of a Doppler sonar go through the same frequency shift during the propagation. Therefore, they share the same Doppler factor. The factor can be easily acquired by the Doppler sonar, and then used to correct CTFM output.

As shown in the verification experiment results, the velocity of the movement keeps on changing, and the Doppler factor value keeps following the speed curve. The corrected range remains very close to the value obtained from the pulse count of the stepper motor, thus proving the validity of the proposed approach. Sample rate and frame size are set at high values in the verification experiments to yield a dense data output. Some other sample rate and frame size values are set in the performance tests. Results show that output precision decreases when velocity is higher. Most mobile agents or indoor targets move at a speed of 1 m/s or lower, with an output range variance reaching 6 × 10^−4^ m^2^. This value is acceptable in most practical applications. As frame size goes larger, output precision goes higher. However, frame size cannot be set too high because the spectral peak of a CTFM sonar output may become vague as a result of the movement.

### 5.2. Complex Target Analysis

In a moving condition, the range profile is affected by Doppler shift and displacement. Doppler shift does not only cause deviation in the range value, but it also stretches or compresses the range profile. The influence of Doppler shift can be reduced by applying the correction approach to the entire profile data. The experimental result of range profile correction can be seen in Figure 13. However, the influence of displacement still needs to be solved.

In this scenario, the frame size should be carefully chosen. A larger frame size provides higher range resolution but makes the range profile fuzzy because of the movement. Assuming that the sonar moves at a certain speed, then a displacement Δ*d* is produced within the frame duration:

(24) $$\Delta d=\frac{vL}{f_{s}}$$

where *L* is the frame size. Frequency resolution of spectrum Δ*f* is the sample rate divided by the frame size. However, a 3 dB dip between two adjacent peaks is required to make them distinguishable [17]. Therefore, the range resolution of the range profile Δ*r* is calculated from Equation (7) using 2Δ*f* such that:

(25) $$\Delta r=\frac{cf_{s}}{mL}$$

By combining the two equations above, a relationship between Δ*d* and Δ*r* can be written as:

(26) $$\Delta d\cdot \Delta r=\frac{cv}{m}$$

Obviously, Δ*d* and Δ*r* are inversely proportional to each other. To maintain an optimized performance, the system needs to adjust frame size *L* to balance between Δ*d* and Δ*r*, according to *v*. For a certain velocity value *v* and a system with a fixed sample rate, the theoretical minimum distinguishable range interval Δ*R* between two targets is the sum of Δ*d* and Δ*r*. According to Equations (24) and (25):

(27) $$\Delta R=\Delta d+\Delta r=\frac{vL}{f_{s}}+\frac{cf_{s}}{mL}$$

**Figure 14.**
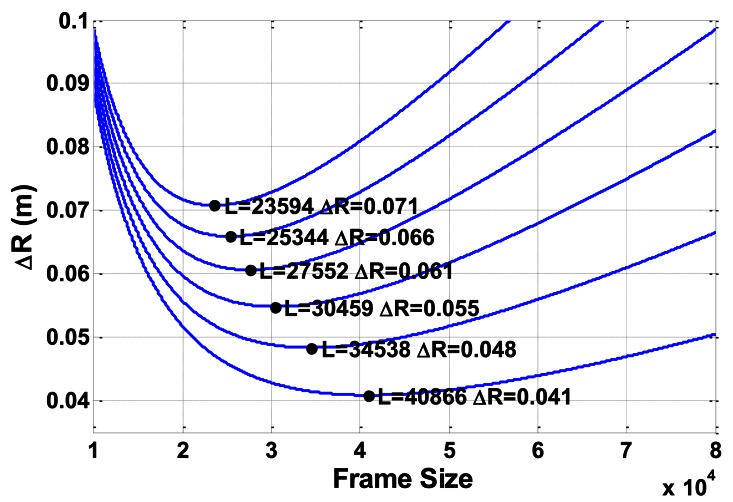
Minimum distinguishable range interval *vs*. frame size.

Curves of Δ*R* on several velocity values are shown in Figure 14. The top one is for *v* = 1.5 m·s^−1^ and the bottom one is for *v* = 0.5 m·s^−1^, with an interval of 0.2 m·s^−1^. For this result, the signal sample rate is set to 1 MS·s^−1^, which is the same as in the previous experiments.

According to Equation (27), a minimum value of Δ*R* exists for a single velocity value. The partial derivative of Δ*R* with respect to *L* is:

(28) $$\frac{\partial \Delta R}{\partial L}=\frac{v}{f_{s}}-\frac{cf_{s}}{mL^{2}}$$

When Equation (28) is set to zero, the value of *L* for the minimum Δ*R* can be written as:

(29) $${L|}_{\min\Delta R}=\text{round}\left(f_{s}\sqrt{\frac{c}{vm}}\right)$$

The minimum points of Δ*R* on each curve are marked as black dots in Figure 14. These minimum values are the finest that the system can reach for the corresponding velocity value. Curves in Figure 14 demonstrate that Δ*R* decreases very fast before reaching the minimum point. After reaching this valley point, Δ*R* increases more slowly. The variation trend of Δ*R* can also be seen in Figure 13.

Note that the higher sample rate does not improve distinguishing ability, and only produces a larger *L* value to reach the minimum Δ*R*. This result can be seen in Equation (29). For a certain velocity, a corresponding boundary of Δ*R* is found:

(30) $$\Delta R_{b}=2\sqrt{\frac{cv}{m}}$$

This finding implies that for a CTFM sonar system on a mobile platform, discrimination ability decreases as movement speed goes higher.

## 6. Conclusions

Although a conventional CTFM sonar is competitive as a ranging sensor, the shortcoming of its erroneous detection under moving conditions results in a great limitation. This work presents an approach to compensate for this flaw and to broaden the application scope of a CTFM sonar. A single tone signal outside the sweep band is added to the CTFM transmitted signal. A Doppler factor can be acquired by extracting the frequency of the single tone component in the echo. Actual target range can be obtained using this Doppler factor and the deviated CTFM output.

Experimental results verify this approach. In this work, data is sampled, framed densely, and then processed in an offline manner. In this manner, output data can be presented with better continuity and higher resolution. A high sample rate and frame rate are actually not necessary in practical applications. Lower sample rates may cause lower resolution of the Doppler factor and the range output. However, as long as it satisfies Nyquist sampling theorem, the proposed approach still does not lose its validity. A low frame rate does not affect the performance of the approach. It can only cause the output of the system to be sparse; however, accuracy is not affected. Therefore, the entire system can be built as an embedded system. The key requirements are a sample rate high enough for ultrasonic signal acquisition and the ability of the processor for FFT analysis. These requirements are the same for a conventional CTFM system. Additional functions are a single tone signal generation and another FFT analysis of the echo signal. These functions do not increase system complexity too much.

With regard to complex targets, the discrimination ability of adjacent reflectors is discussed. Doppler shift and displacement both affect range profile. The choice of frame size is a key point to allow better separation of adjacent targets.

This research provides a basic idea for applying a CTFM sonar in detections involving moving conditions. Further research will include the following aspects: (1) implementation of the approach as a real-time system; (2) moving target recognition using the range profile data obtained through the approach described in Subsection 5.2; translation-invariant feature extraction methods and suitable recognition methods should also be investigated; and (3) the development of a multiple target detection ability; the matching problem between Doppler factors and CTFM outputs needs to be solved.

## Figures and Tables

**Table 1.** Signal parameter setup of the experiments.

*f_H_*	*f_L_*	*T_sw_*	*m*	*f_ST_*
80 kHz	40 kHz	0.1 s	400 kHz/s	100 kHz
